# Cognitive and Sensory Dimensions of Older People’s Preferences of Outdoor Spaces for Walking: A Survey Study in Ireland

**DOI:** 10.3390/ijerph16081340

**Published:** 2019-04-14

**Authors:** Marica Cassarino, Eleanor Bantry-White, Annalisa Setti

**Affiliations:** 1 School of Applied Psychology, University College Cork, Enterprise Centre, North Mall, T23 TK30 Cork City, Ireland; a.setti@ucc.ie; 2 School of Applied Social Studies, University College Cork, William Thompson House, Donovan’s Road, T12 E6F3 Cork City, Ireland; e.bantrywhite@ucc.ie

**Keywords:** outdoor spaces, aging, physical activity, sensory sensitivity, cognitive failures

## Abstract

Background: Physical exercise, particularly walking, benefits healthy ageing. Understanding the environmental circumstances in which exercise occurs is crucial to the promotion of physical activity in older age. Most studies have focused on the structural dimensions of environments that may foster walking; however, individual differences in how older people perceive and interact with outdoor spaces need further attention. This study explored the cognitive and sensory dimensions of preferences of outdoor spaces for walking. Methods: We invited 112 healthy community-dwelling people aged ≥60 years to complete a survey to test associations between walking preferences and cognitive/sensory vulnerability. A subsample also completed focus groups/walk along interviews to explore qualitatively the cognitive/sensory reasons for outdoor walking preferences. Results: While most participants indicated a preference for outdoor spaces that offer variety and greenery, we observed a complex association between individual cognitive/sensory needs (stimulation seeking vs. avoidance), preferences for social interactions, and the place of residence urbanity level. Furthermore, walking preferences varied based on the purpose of the walk (recreation vs. transportation). Conclusions: Our findings support an ecological approach to understanding determinants of physical activity in older age, which consider the interaction between individual cognitive processing and the environment.

## 1. Introduction

Engaging in physical activity, particularly walking, has been associated with significant benefits for health and wellbeing in older age [1,2,3,4]. For this reason, it is crucial to identify the factors that promote the involvement of older people in physical activity [5]. Within this research, the role of the outdoor built environment has increasingly been investigated to clarify which places can facilitate active lifestyles, particularly in older age [6,7,8,9]. While research has advanced in capturing determinants of walkability [7], recent investigations suggest that in order to understand what environmental circumstances favour active lifestyles, it is crucial to examine characteristics of both individuals and the environment [10]; this person-environment fit approach was developed within ecological models of ageing [11]. A concept that has become central in the investigation of person-environment relationships is that of usability [12]; as described by Iwarsson and Ståhl [12], usability refers to the ability of the individual to interact with an environment according to their needs and goals. Considering influences of outdoor physical activity in older age from the perspective of usability requires an integrated investigation of three dimensions: (a) the individual with their physical and mental needs/strengths; (b) the environment with its enablers and barriers; (c) the specific interaction between a person and their surroundings, that is, the activities performed by an individual in a certain environment [12].

Structural and physical characteristics of the environment, indeed, affect walking behaviour [13]. However, outdoor spaces do not necessarily present enablers or barriers per se but provide more or fewer opportunities for activities based also on the characteristics of the individual. Experiential perspectives on walking have gained interest and individuals’ perspectives on social and structural determinants of physical activities have been increasingly investigated [14]; despite these advancements, the cognitive and perceptual dimensions of preferences of outdoor spaces for walking are poorly understood, particularly in older people [15]. Clarifying the cognitive and perceptual dimensions of usability is crucial to understanding physical activity habits, such as walking. Emerging research has shown that the built environment influences perceptual and mental processing, which in turn affects behaviour [16,17,18,19,20,21,22]. Furthermore, accumulating studies have demonstrated that sensory or cognitive vulnerability in older age is associated with altered walking behaviour [23,24]. In light of this recent literature, there is a need to enrich our understanding of the cognitive and perceptual dimensions of environmental influences on walking in older age.

In this paper, we explored whether individual cognitive and/or sensory characteristics were associated with variations in preferences of outdoor spaces for walking in older age by means of a survey (“Outdoor Lived Environment as Brain Training”, or “OutLET”, survey). With reference to the framework on usability proposed by Iwarsson [12], we investigated person-environment relationships by looking at how walking preferences were influenced by the interaction between individuals’ cognitive and sensory vulnerability and the physical and social characteristics of the environment. We also conducted focus groups and walk along interviews with a subgroup of our participants to enrich our understanding of survey responses through a qualitative exploration of the subjective experiences of interacting with outdoor spaces. Our working hypothesis was that older people choose outdoor spaces that meet their stimulation needs: Individuals with higher levels of cognitive problems/sensory sensitivity would perceive more stressors and barriers to active lifestyles in the outdoors than individuals with lower sensory/cognitive issues [20] and thus prefer environments with lower levels of stimulation. These preferences, however, could also change based on the level of stimulation afforded by the environments that people can access, for example in terms of level of urbanity or green [22].

## 2. Methods

### 2.1. Participants

A sample of 112 community-dwelling people aged ≥60 years in the Southern region of Ireland completed the “OutLET” survey, and 28 of these took also part in focus groups (*n* = 21) or 1:1 walk-along interviews (*n* = 7). Participants were recruited through convenience and snowball sampling: We contacted active retirement groups and individual senior citizens. All participants read an information sheet and signed a consent form prior to participation. The study was conducted in accordance with the Declaration of Helsinki and received ethical approval (see Supplementary File 1) in September 2017 from the School of Applied Psychology Ethics Committee at University College Cork (Ireland).

### 2.2. Design

In this observational study, all participants completed a survey designed to test associations between cognitive/sensory characteristics and preferences of outdoor spaces for walking. A subsample of participants was also interviewed to explore these associations qualitatively.

### 2.3. Survey

The OutLET survey was developed by drawing from the Age Friendly Cities criteria [25], the academic literature on walkability [7], cognitive restoration [26] and sensory sensitivity [27]. The survey includes five sections that investigate characteristics of the individual (socio-demographic, health, sensory and cognitive sensitivity) and of the environments where they reside and/or which they use for outdoor activities (including structural and social circumstances). The survey is available both online and in a pen-and-paper version; it can be completed by the participant alone or used as a structured interview by the experimenter. A detailed publication on the development and piloting of the survey is currently in preparation. The measures used for the purpose of this study are described below.

#### 2.3.1. Outcome Measures

Preferences of outdoor environments for walking were assessed through four items representing environmental dimensions that have been shown in the literature to be associated with cognitive and sensory processing: Variety of things to see, also defined as complexity [22], quietness [23], green spaces [26], and presence of people as a measure of crowding [28]. Participants were asked to rate the importance of having these aspects in the outdoor places where they walk (from 1 “not at all” to 5 “very much”). A subsample of 60 participants were also asked to indicate their frequency of walking in the neighbourhood of the residence or other places for recreation (e.g., exercise, walking a pet) or transportation (e.g., shopping).

#### 2.3.2. Independent Measures

Independent measures included cognitive failures and sensory sensitivity. Cognitive vulnerability was assessed using the Cognitive Failure Questionnaire, or CFQ [29]. This is a 25-item questionnaire that asks a person to rate the frequency with which cognitive failures have occurred to them in the previous six months (from 0 “Never” to 4 “Very often”). As described by Bridger et al. [30], the CFQ measures “the incidence of critical mismatches between cognitive control processes and the external environment” (p. 7). While there is inconsistency in the literature about the factorial structure of the CFQ [30,31], the one factor solution suggested by Broadbent [29] has been employed widely and the instrument has demonstrated good predictive and criterion validity, as well as relative stability over time [30]. We created a cognitive failure score by adding up the scores of the 25 items (Cronbach’s alpha = 0.88), with higher scores indicating a higher frequency of cognitive failures in day-to-day activities.

Sensory sensitivity was assessed through 11 questions measuring the individual’s level of sensitivity to sensory stimulation coming from the surrounding environment (on a 5-point Likert scale from 1 “Very much unlike me” to 5 “Very much like me”); the questionnaire was created using items from existing measures of sensory processing usually applied to individuals with autistic characteristics but also transferable to the general population [27,32,33]. The scores for each item were added up to create a total sensory sensitivity score ranging from 11 to 55 (Cronbach’s alpha = 0.76), with a higher score indicating a higher sensory sensitivity.

#### 2.3.3. Covariates

Our analyses controlled for the level of urbanity of the place of residence, as previous studies have observed urban-rural variations in walking/physical activity preferences [34,35]: to this end, we asked survey respondents to describe the level of urbanity of their neighbourhood of residence (1 “inner city” 2 “city suburbs” 3 “town” 4 “village” 5 “countryside”); these categories were based on the categorisation adopted by the Irish Central Statistics Office (www.cso.ie). In the survey, the respondents were advised to consider “neighbourhood” as the area around their house that they could cover by walking for 15 min. While the respondents self-rated the level of urbanity of their place of residence, we randomly checked subjective ratings using the neighbourhood name/address provided by the participants (captured in the survey, but not reported here) for 10% of the data and found that self-ratings matched the Census categorisation.

Socio-demographic covariates included gender (male or female) and age in years. While education, employment, and health status were assessed in the survey, these measures were not included in the analyses because the data was highly skewed towards highly educated and retired individuals, and most of the participants rated their health as very good or excellent.

To check whether walking preferences varied based on walking habits, we also assessed frequency of walking (from 1 “Never” to 5 “Every day”) and company preferences while walking (alone, in company, or no preference).

### 2.4. Data Analysis

Analyses of the survey data were conducted using IBM SPSS v.24 (IBM, Armonk, NY, USA). The dataset is publicly available at: https://doi.org/10.17605/OSF.IO/8NP73. Descriptive statistics (frequencies and percentages for categorical measures, mean, standard deviation, or median and interquartile range for continuous measures) were used to provide an overview of the sample characteristics in relation to the measures of interest. We investigated bivariate associations between the measures of interest by using Spearman’s correlation for continuous variables (including the outcome and independent measures, walking frequency, and age); we used the Mann-Whitney test to explore gender-based differences in walking preferences, and the Kruskal-Wallis test to compare walking preferences among participants living in areas with different levels of urbanity and also based on company preferences when walking. The effects of cognitive failures and sensory sensitivity on walking preferences were separately tested in interaction with the level of urbanity of the place of residence using ordinal logistic regression with proportional odds ratios.

The exploratory interviews and focus groups were audio-recorded, transcribed, and entered into the Qualitative data analysis software NVivo 11 Pro to conduct a thematic analysis. An inductive approach to analysis, linked to the research question, was undertaken. In accordance with Braun and Clark [36,37], we adhered to six stages of analysis; two researchers (MC and EBW) independently: (1) familiarised themselves with the data, (2) identified codes, and (3) sorted codes into themes; (4) the themes were then compared and reviewed jointly to ensure homogeneity; (5) the two authors named and defined the themes together and (6) jointly generated the underlying story linking the themes. Disagreements between the two researchers were solved by consensus. As the qualitative part of the study was exploratory and conducted on a subsample, the themes are briefly described in the Results and used in the Discussion to enrich our understanding of the quantitative findings. Detailed information on the themes and extracts is presented in Supplementary File 2.

## 3. Results

### 3.1. Sample Characteristics

A detailed breakdown of sample characteristics is included in Supplementary File 3. In this sample (Mean age = 70.55, SD = 8.63; 64.3% female), participants reported overall intermediate levels of sensory sensitivity (Mean = 30.57, SD = 8.28) and intermediate frequency of cognitive failures (Mean = 31.66, SD = 13.35). Considering preferences of outdoor places for walking, most participants indicated variety, quietness, and green spaces as important, whereas ratings on presence of people showed heterogeneity of responses across the sample. Over half (58%) of the participants rated their place of residence as urban (inner city, city suburbs or town) and 41% as rural (village or countryside). In terms of walking habits, the majority of the sample (67%) reported walking every day or quite often; 38.4% of the sample reported no preferences between walking alone or in company, but 32.1% preferred walking alone.

### 3.2. Factors Associated with Walking Preferences

We initially conducted bivariate analyses to test associations between cognitive/sensory measures and walking preferences. As shown in Table 1, participants who reported a higher frequency of cognitive failures were less inclined to walk in places characterised by presence of people (*rho* = −0.25, *p* = 0.02), but no significant associations emerged between cognitive failures and any of the other walking preferences. On the other hand, sensory sensitivity did not correlate significantly with any of the walking preferences. The variables of variety, quietness, and green spaces appeared to be all positively associated with each other, with the correlation coefficients indicating that these variables measured distinct but related constructs. Conversely, the presence of people was not associated with any of the other walking preferences. Similarly, cognitive failures and sensory sensitivity did not correlate significantly with each other, indicating that the two variables measured separate constructs.

Considering variations in walking preferences based on level of urbanity of the place of residence, no differences were noted for variety (*Chi2*_4_ = 1.62, *p* = 0.81), quietness (*Chi2*_4_ = 3.87, *p* = 0.43), or green spaces (*Chi2*_4_ = 3.62, *p* = 0.46), but significant differences emerged for presence of people (*Chi2*_4_ = 13.03, *p* = 0.011): Participants living in the inner city assigned the lowest ratings of importance to this aspect of outdoor spaces (median = 2.00, *IQR* = 2.00) compared to the other groups (suburbs: median = 4.00, *IQR* = 2.00; towns: median = 4.00, *IQR* = 2.00; village: median = 4.00, *IQR* = 3.00; countryside: median = 3.00; *IQR* = 2.00).

In terms of other factors, we observed that frequent walkers rated green spaces as important aspects of the places where they walk (*rho* = 0.21, *p* = 0.04) and a trend towards significance emerged also for presence of people (*rho* = 0.18, *p* = 0.06), as shown in Table 1. No associations between walking preferences and age were noted. On the other hand, a Mann-Whitney test showed that women rated presence of people as significantly more important than men (*Z* = −2.64, *p* = 0.008; Male: median = 3.00, *IQR* = 2.00; Female: median = 4.00, *IQR* = 2.00); no gender-based differences emerged for variety (*Z* = −0.28, *p* = 0.78), quietness (*Z* = −1.15, *p* = 0.25), or green spaces (*Z* = −0.03, *p* = 0.97). We also checked for variations in environmental preferences for walking based on company preferences and found that those who preferred walking in company reported the highest preference for the presence of other people (*Chi2*_2_ = 6.96, *p* = 0.03) and the lowest preference for quietness (*Chi2*_2_ = 7.73, *p* = 0.02), whereas no significant differences emerged for variety (*Chi2*_2_ = 3.63, *p* = 0.16) or green spaces (*Chi2*_2_ = 1.59, *p* = 0.45).

Ordinal logistic regressions were used to test the synergistic influence of cognitive/sensory characteristics and urbanity levels on environmental preferences for walking while controlling for age and gender. As shown in Table 2, a significant interaction between cognitive failures and level of urbanity of the place of residence emerged for the presence of people, whereby participants who reported a higher frequency of cognitive failures and lived in inner city, villages, or the countryside, but not city suburbs or towns, showed a significantly lower likelihood of preferring the presence of people. A similar pattern emerged for sensory sensitivity (Table 2): Increasing self-reported sensory sensitivity was significantly associated with a lower likelihood of walking in places characterised by the presence of people for participants living in the inner city and the countryside. The regression models were conducted also for variety, quietness and green spaces but were not significant (Supplementary File 4).

### 3.3. Walking Destinations and Types

In the subsample of 60 participants who gave information on their specific walking habits, around 60% reported recreational walking in their neighbourhood (e.g., exercise, walking a pet) quite often or every day, and over a third (38%) used the neighbourhood for transportation walking (i.e., day-to-day activities such as shopping). At the same time, some participants walked also in places other than their neighbourhood on a regular basis (33.9% recreation; 32.1% transportation).

Walking purpose or destination were not associated with cognitive failures or sensory sensitivity. Recreational walking in the neighbourhood was associated with variations in walking preferences: Participants who walked in their neighbourhoods for recreational reasons preferred more to walk in places with green spaces (*rho* = 0.34, *p* = 0.008) and people (*rho* = 0.31, *p* = 0.02), but correlations, although positive, were not significant for variety (*rho* = 0.14, *p* = 0.28) or quietness (*rho* = 0.21, *p* = 0.12). Furthermore, no significant correlations emerged between walking preferences and other walking types (recreational walking outside the neighbourhood, or transportation walking in or outside the neighbourhood). Walking destinations and types did not vary by urbanity level, although a trend towards significance was found for transportation walking in the neighbourhood (Kruskal Wallis *Chi2*_4_ = 9.43, *p* = 0.051), whereby participants living in towns reported the highest frequency (median = 5.00, *IQR* = 0.00) and those living in the countryside reported the lowest (median = 1.00, *IQR* = 2.00). No other variations in environmental use were noted based on location type.

In terms of individual factors, women reported recreational walking in their neighbourhood more than men, although these differences showed only a trend towards significance (*Z* = −1.94, *p* = 0.05; Male: median = 3.00, *IQR* = 4.00; Female: median = 4.00, *IQR* = 4.00); there were no significant gender-based differences for other types of use (neighbourhood transportation: *p* = 0.44; other places recreation: *p* = 0.59; other places transportation: *p* = 0.72).

### 3.4. Qualitative Themes

The group of participants who completed the qualitative part of the study (*n* = 28), had a mean age = 72.00 (*SD* = 7.72), with 82.14% female participants. Most of the sample (82.2%) reported to live in an urban neighbourhood (inner city, city suburbs or towns). The frequency of walking varied among participants, with some doing it daily and others less frequently; the participants who took part in the walking interviews (*n* = 7) were regular walkers.

The conversations held with the participants highlighted great diversity and heterogeneity of experiences. Five main themes denoted individual differences in choice and use of outdoor spaces (see Supplementary File 2 for a detailed description and extracts):

(1) Diversity of walking purposes: The choice of outdoor spaces was determined by the purpose of the walk (recreation vs. transportation) and also by the level of urbanity of the place of residence: urban dwellers walked for transport in their neighbourhood but drove to green/blue spaces for recreation, with the opposite pattern for rural dwellers;
(2) Stimulation adjustment needs: Within-person variations in environmental preferences were observed based on the level of mental stimulation sought by the individual; stimulation seekers looked for places offering variety and novelty, such as the city centre, whereas natural/green spaces were preferred if the person wanted to avoid or reduce stimulation.
(3) Personal attitudes towards outdoor spaces: Together with transitory stimulation adjustment needs, environmental preferences varied based on personal attitudes towards stimulation, particularly in terms of social interactions: Individuals who sought social engagement preferred places used by other people and walking in company, whereas individuals who preferred to avoid social encounters used less crowded places and natural spaces. Biophilia and sense of attachment to places further contributed to between-person variations in outdoor preferences.
(4) Social dimensions of walking outdoors: Participants’ views on the presence of other people in the places where they walk were also associated with their perceptions of social safety: Having people around made some participants feel as being in a safe environment, but these feelings varied based on the types of people that could be encountered outdoors.
(5) Physical attributes of outdoor spaces: In line with previous studies on walkability, our participants also discussed the important role of the environment structural quality and accessibility of outdoor spaces for choosing to walk outdoors.

## 4. Discussion

### 4.1. Summary of Findings

In this study we explored links between individuals’ cognitive/sensory characteristics and preferences of outdoor spaces for walking in a sample of healthy older adults while also considering the level of urbanity of their place of residence.

In our analyses we observed an interesting interaction whereby individuals in very urbanised or very rural places, who reported higher cognitive failures or sensory sensitivity, expressed a lower preference for the presence of people in the places where they walk. Previous studies have observed a negative impact of crowding on cognitive performance in older adults [28]. Further, accumulating evidence supports the idea that nature can have restorative effects on cognition [19] and urban environments have been linked to social stress [38]. However, our study seems to suggest that environmental characteristics can influence preferences in synergy with the stimulation needs of the individual. This view is supported by themes 2–4, which emerged in the interviews. When discussing their preferences, our participants described choosing outdoor spaces for walking based on their transitory stimulation needs, their personal attitudes towards social stimulation, and their views on social safety. As observed in Theme 2, being in need of stimulation would bring the person to busy (urban) places, whereas stimulation avoidance led to walking in more isolated (and natural) places. Furthermore, individual attitudes towards social stimulation (seeking vs. avoiding) guided the choice of using urban or natural spaces (Theme 3): For a number of participants the presence of people in outdoor spaces was seen as a motivator to go out in busy places, to facilitate social interactions. Conversely, some participants actually preferred natural spaces to avoid other people because people were perceived as a source of stress. Lastly, participants indicated that their preference for the presence of people when walking outdoors depended on how safe those people were perceived (Theme 4) based on activity and outfit, but also based on location (e.g., inner city or isolated walks were associated with a higher fear of crime). The presence and activities of people encountered when outdoors has been shown to affect the level of perceived attractiveness of outdoor spaces for walking [39,40]. What appears to emerge from our study is that having people around is not only a social dimension of walking, but also represents an indicator of the level of stimulation afforded by the environment. In support of this, the extant literature has shown both positive and negative effects of social aspects the environment on wellbeing: Being in an environment that fosters relationships can benefit older people’s wellbeing [41], and studies have observed the benefits of “social walking” [9], but at the same time crowding has been linked to reduced comfort of movement [42]. In our study, a negative association between cognitive vulnerability and preference for crowded places was observed for participants in both very urbanised and very rural places, indicating that the level of urbanity of the places of residence can also contribute to how people perceive social stimulation in the outdoors. On one hand, urbanised places, such as inner cities, can easily cause mental overload [38], and thus be more stressful for individuals with cognitive vulnerability. On the other hand, based on the literature on cognitive restoration in nature [19], living in the countryside with ease of access to nature could lead people to have a lower threshold for the cognitive demands imposed by the environment, such as crowding, as well as a higher sense of biophilia, which in the interviews was associated with preference for walking alone (Theme 3). As discussed elsewhere [22], different levels of urbanity can afford varying levels of cognitive stimulation, but whether that stimulation is optimal depends also on the cognitive characteristics of the person, as increasingly acknowledged in research on person-environment relationships [43]. Thus, our findings suggest that the individual’s cognitive characteristics and attitudes towards social stimulation, together with characteristics of the outdoor environment, can play an important role in determining whether the person might or might not benefit mentally from walking in places used by other people.

In addition, we observed gender-based differences in walking preferences for social interactions, which have been demonstrated in the extant literature [44]. Studies suggest that older women might benefit more than men in terms of wellbeing from experiencing positive social exchanges [45]. These differences, however, need further investigation in relation to walking behavior, as a recent systematic review noted that the social dimension of walking has been investigated more in women than in men [46].

Considering other walking preferences, the lack of significant associations between cognitive/sensory characteristics and variety or green spaces might be due to a ceiling effect, whereby the majority of our participants indicated preference for these two aspects of outdoor places. In the interviews, some participants expressed preference for walking in places that offer easy access to both variety and green spaces, such as urban green areas. The benefits for health and wellbeing of having green areas in the city have been increasingly shown in research [47] and the restorative effects of nature on cognition are well-established [19]. An interesting qualitative finding was that biophilia influenced the choice of walking in nature rather than other places (Theme 3); nature orientation has been observed as a stronger predictor of the preference to walk in natural settings than the mere availability of green spaces [48], once again highlighting the pivotal role of individual attitudes and goals on environmental preferences. However, we were unable to fully explore this aspect as a potential moderator due the absence of an item on biophilia in the survey. Importantly, we cannot conclude from our data whether all participants considered green spaces the same way (e.g., urban park vs. wild nature), which highlights the need for a more structured operationalization of “green”.

Quietness was also rated as important by the majority of the participants; although we found no associations with cognitive/sensory measures, the need for quietness was expressed in the survey particularly by those participants who liked to walk alone, whereas people who liked walking in company rated the presence of people as more important than quietness. Moreover, in the interviews, participants described staying away from noisy or crowded places if in need of avoiding stimulation. This finding speaks once again to the role of social stimulation for walking outdoors, and indeed, noise has been found to negatively affect cognitive performance [23], but further analyses with a more varied sample are needed to define clear links with cognitive needs.

While we did not explore structural quality (accessibility, aesthetic appeal) and safety in the survey, these factors appeared to influence the choice of outdoor spaces in the interviews/focus groups (Theme 5). These findings are in line with previous qualitative investigations [49,50], confirming that both social and structural characteristics of outdoor environments are important contributors to individuals’ preferences for walking, and will inform future iterations of the survey.

In a smaller section of our sample, the purpose of the walk (recreational vs. transportation) emerged as an important contributor to the decision of walking in the neighbourhood of residence or other places both in the survey and the interviews. The purpose of the walk did not appear to be associated with cognitive failures or sensory sensitivity, but participants who had a higher preference for green spaces and the presence of people walked more frequently in their neighbourhood for recreation. Whether these ratings depended on the availability of green spaces and people in the neighbourhood of residence remains unclear from our data and deserves further investigation (i.e., are older people more likely to walk in their neighbourhood if there are green spaces and opportunities for social interactions?). Interestingly, the frequency of transportation walking in the neighbourhood of residence varied based on level of urbanity: Rural participants reported lower levels of transportation walking in their neighbourhood than urban residents, but no patterns emerged for recreational walking, probably due to the fact that the majority of the participants reported recreational walking in their neighbourhood independent of where they lived. Urban-rural variations in recreational and transportation walking among older people have been observed in previous studies [34,35] and could be linked to the accessibility of services/amenities. Our findings are, however, exploratory, and investigations of walk purpose with bigger and more varied samples are needed to clarify potential links with cognitive and environmental characteristics.

### 4.2. Strengths and Limitations

The main strength of our study is its novel focus on cognitive and sensory dimensions of interacting with outdoor environments, for which limited evidence is currently available [20]. This study, then, enriches existing ecological frameworks of outdoor usability. Also, integrating qualitative data with the findings of the survey enabled us to gain a richer understanding of associations between cognitive characteristics and outdoor preferences for walking.

Our findings must, however, be interpreted in light of some limitations. Our small sample size, the high educational and health status of our participants, and the fact that all participants lived in the same geographical area limit the generalisability of our results. In addition, while we did not assess area socio-economic status objectively, our sample was quite homogeneous in that most individuals lived in affluent areas or at least not deprived areas. These factors, together with the convenience sampling adopted, introduce a potential selection bias and call for further investigations involving people from a variety of socio-economic backgrounds, as area socio-economic status has been shown to influence walking behaviour [8]. Also, while the interviews provided interesting insights about the influence of the subjective need for social stimulation on outdoor preferences for walking, they were conducted with a smaller sample of participants, and thus the complex associations emerged need further study, employing a robust mixed-methods design. Despite these limitations, we observed similar patterns of walking preferences to those of previous studies, for example, in terms of the influence of structural and social characteristics of the environment on walking [49]. Lastly, we investigated environmental preferences for walking using measures that have demonstrated links with cognitive and sensory status, but further measures should be explored, like, for example blue spaces, which have increasingly been linked to mental wellbeing, as well as physical activity [51].

## 5. Conclusions

Our study explored the cognitive and sensory dimensions of walking preferences in the outdoors with a sample of healthy older individuals. Our findings indicate that the way in which individuals perceive social interactions in the places where they walk influence preferences of outdoor spaces for walking; their perceptions, on the other hand, appeared to be dictated by the person’s cognitive and sensory attitudes towards external stimulation as well as environmental circumstances. Although this is a cross-sectional study, it highlights the importance of taking into account walking preferences and whether they are met by the lived environment, in relation to the expected cognitive benefits of walking. This is particularly necessary for those who report cognitive failures and may benefit from walking more if the preferences are easily met in the available surroundings.

## Figures and Tables

**Table 1:** Nonparametric correlations.

Measure	1	2	3	4	5	6	7
1. Variety							
2. Quietness	0.42 ***						
3. Green spaces	0.32 ***	0.47 ***					
4. People	0.14	−0.17	0.12				
5. Cognitive failures	0.03	0.02	−0.09	−0.250 *			
6. Sensory sensitivity	0.14	0.18	0.03	−0.13	0.17		
7. Walking frequency	−0.04	0.05	0.21 *	0.180 ^†^	−0.04	−0.190 *	
8. Age	−0.02	0.06	−0.13	0.05	0.240 *	0.270 **	0.04

Notes: Estimates represent *rho* coefficients of Spearman’s correlation. Statistical significance is presented as * *p* < 0.05, ** *p* < 0.01, *** *p* < 0.001, † *p* < 0.07 (trend).

**Table 2:** Preference for the Presence of People based on Cognitive/Sensory Measures and Urbanity Level.

Measure	Cognitive Failures			Sensory Sensitivity		
	Prop OR	*p*-Value	Wald *Chi2*	Prop OR	*p*-Value	Wald *Chi2*
Cognitive failures by urbanity level			17.12 **			15.52 **
Inner city	0.93	0.003		0.92	0.01	
City suburbs	0.98	0.34		0.98	0.62	
Town	0.99	0.91		0.99	0.75	
Village	0.96	0.06		0.97	0.34	
Countryside	0.95	0.004		0.94	0.03	
Female	3.15	0.009	6.86 **	3.12	0.007	7.32 **
Age	1.01	0.97	0.01	0.99	0.64	0.22
Goodness-of-fit Pearson’s chi2	393.66			381.39		
Omnibus test likelihood ration chi2	23.45	0.001		22.34	0.002	
Pseudo *R^2^*	0.08			0.07		

*Notes*. *Chi2* = Chi-squared; Female refers to the Gender variable, with female participants compared to male participants; Prop OR = Proportional Odds Ratio. Model 2 controls for gender and age. Wald chi2 refers to the test of model effects. Statistical significance for this test is shown as * *p* < 0.05, ** *p* < 0.01, *** *p* < 0.001.

## Supplementary Materials

The following are available online at https://www.mdpi.com/1660-4601/16/8/1340/s1, Supplementary File 1: Ethics approval; Supplementary File 2: Qualitative themes and Interview schedule: Supplementary File 3: Sample Characteristics; Supplementary File 4: Regression analyses.

## Dataset Identifier

https://doi.org/10.17605/OSF.IO/8NP73.

## References

[1] Kelly M.E., Loughrey D., Lawlor B.A., Robertson I.H., Walsh C., Brennan S. (2014). The impact of exercise on the cognitive functioning of healthy older adults: A systematic review and meta-analysis. Ageing Res. Rev..

[2] Aarsland D., Sardahaee F.S., Anderssen S., Ballard C. (2010). Alzheimer’s Society Systematic Review group Is physical activity a potential preventive factor for vascular dementia? A systematic review. Aging Ment. Health.

[3] Murtagh E.M., Nichols L., Mohammed M.A., Holder R., Nevill A.M., Murphy M.H. (2015). The effect of walking on risk factors for cardiovascular disease: An updated systematic review and meta-analysis of randomised control trials. Prev. Med..

[4] Korpela K., Borodulin K., Neuvonen M., Paronen O., Tyrväinen L. (2014). Analyzing the mediators between nature-based outdoor recreation and emotional well-being. J. Environ. Psychol..

[5] Koeneman M.A., Verheijden M.W., Chinapaw M.J.M., Hopman-Rock M. (2011). Determinants of physical activity and exercise in healthy older adults: A systematic review. Int. J. Behav. Nutr. Phys. Act..

[6] Brownson R.C., Hoehner C.M., Day K., Forsyth A., Sallis J.F. (2009). Measuring the Built Environment for Physical Activity. State of the Science. Am. J. Prev. Med..

[7] Kerr J., Rosenberg D., Frank L. (2012). The Role of the Built Environment in Healthy Aging: Community Design, Physical Activity, and Health among Older Adults. J. Plan. Lit..

[8] Annear M., Keeling S., Wilkinson T., Cushman G., Gidlow B., Hopkins H. (2014). Environmental influences on healthy and active ageing: A systematic review. Ageing Soc..

[9] Chaudhury H., Campo M., Michael Y., Mahmood A. (2016). Neighbourhood environment and physical activity in older adults. Soc. Sci. Med..

[10] McCormack G.R., Shiell A. (2011). In search of causality: A systematic review of the relationship between the built environment and physical activity among adults. Int. J. Behav. Nutr. Phys. Act..

[11] Lawton M., Nahemow L. (1973). Ecology and the aging process: Psychology of adult development and aging. Psychology of Adult Development and Aging.

[12] Iwarsson S., Ståhl A. (2003). Accessibility, usability and universal design—Positioning and definition of concepts describing person-environment relationships. Disabil. Rehabil..

[13] Ghani F., Rachele J.N., Loh V.H., Washington S., Turrell G. (2018). Do differences in built environments explain age differences in transport walking across neighbourhoods?. J. Transp. Health.

[14] Franco M.R., Tong A., Howard K., Sherrington C., Ferreira P.H., Pinto R.Z., Ferreira M.L. (2015). Older people’s perspectives on participation in physical activity: A systematic review and thematic synthesis of qualitative literature. Br. J. Sports Med..

[15] Withagen R., de Poel H.J., Araújo D., Pepping G.J. (2012). Affordances can invite behavior: Reconsidering the relationship between affordances and agency. New Ideas Psychol..

[16] Coburn A., Vartanian O., Chatterjee A. (2017). Buildings, beauty, and the brain: A neuroscience of architectural experience. J. Cogn. Neurosci..

[17] Tilley S., Neale C., Patuano A., Cinderby S. (2017). Older people’s experiences of mobility and mood in an urban environment: A mixed methods approach using electroencephalography (EEG) and interviews. Int. J. Environ. Res. Public Health.

[18] Neale C., Aspinall P., Roe J., Tilley S., Mavros P., Cinderby S., Coyne R., Thin N., Bennett G., Thompson C.W. (2017). Correction to: The Aging Urban Brain: Analyzing Outdoor Physical Activity Using the Emotiv Affectiv Suite in Older People. J. Urban Health.

[19] Ohly H., White M.P., Wheeler B.W., Bethel A., Ukoumunne O.C., Nikolaou V., Garside R. (2016). Attention Restoration Theory: A systematic review of the attention restoration potential of exposure to natural environments. J. Toxicol. Environ. Health Part B Crit. Rev..

[20] Ojala A., Korpela K., Tyrväinen L., Tiittanen P., Lanki T. (2019). Restorative effects of urban green environments and the role of urban-nature orientedness and noise sensitivity: A field experiment. Health Place.

[21] Brookfield K., Thompson C.W., Scott I. (2017). The uncommon impact of common environmental details on walking in older adults. Int. J. Environ. Res. Public Health.

[22] Cassarino M., Setti A. (2016). Complexity as key to designing cognitive-friendly environments for older people. Front. Psychol..

[23] Setti A., Burke K.E., Kenny R.A., Newell F.N. (2011). Is inefficient multisensory processing associated with falls in older people?. Exp. Brain Res..

[24] Paraskevoudi N., Balcı F., Vatakis A. (2018). “Walking” through the sensory, cognitive, and temporal degradations of healthy aging. Ann. N. Y. Acad. Sci..

[25] Gibney S., Shannon S. (2018). Developing indicators for the age-friendly cities and counties programme in Ireland. Work. Older People.

[26] Kaplan S. (1995). The restorative benefits of nature: Toward an integrative framework. J. Environ. Psychol..

[27] Robertson A.E., Simmons D.R. (2013). The relationship between sensory sensitivity and autistic traits in the general population. J. Autism Dev. Disord..

[28] Merriman N.A., Ondřej J., Rybicki A., Roudaia E., O’Sullivan C., Newell F.N. (2018). Crowded environments reduce spatial memory in older but not younger adults. Psychol. Res..

[29] Broadbent D.E., Cooper P.F., FitzGerald P., Parkes K.R. (1982). The Cognitive Failures Questionnaire (CFQ) and its correlates. Br. J. Clin. Psychol..

[30] Bridger R.S., Johnsen S.Å.K., Brasher K. (2013). Psychometric properties of the Cognitive Failures Questionnaire. Ergonomics.

[31] Craig Wallace J. (2004). Confirmatory factor analysis of the cognitive failures questionnaire: Evidence for dimensionality and construct validity. Pers. Individ. Differ..

[32] Brown C., Tollefson N., Dunn W., Cromwell R., Filion D. (2001). The adult sensory profile: Measuring patterns of sensory processing. Am. J. Occup. Ther..

[33] Blanche E.I., Parham D., Chang M., Mallinson T. (2014). Development of an adult sensory processing scale (ASPS). Am. J. Occup. Ther..

[34] Cleland V., Sodergren M., Otahal P., Timperio A., Ball K., Crawford D., Salmon J., McNaughton S.A. (2015). Associations between the Perceived Environment and Physical Activity among Adults Aged 55–65 Years: Does Urban-Rural Area of Residence Matter?. J. Aging Phys. Act..

[35] Van Cauwenberg J., De Bourdeaudhuij I., De Meester F., Van Dyck D., Salmon J., Clarys P., Deforche B. (2011). Relationship between the physical environment and physical activity in older adults: A systematic review. Health Place.

[36] Braun V., Clarke V. (2006). Using thematic analysis in psychology. Qual. Res. Psychol..

[37] Braun V., Clarke V., Terry G. (2012). Thematic analysis. Qual. Res. Clin. Heal. Psychol..

[38] Lederbogen F., Kirsch P., Haddad L., Streit F., Tost H., Schuch P., Wüst S., Pruessner J.C., Rietschel M., Deuschle M. (2011). City living and urban upbringing affect neural social stress processing in humans. Nature.

[39] Sugiyama T., Francis J., Middleton N.J., Owen N., Giles-Corti B. (2010). Associations between recreational walking and attractiveness, size, and proximity of neighborhood open spaces. Am. J. Public Health.

[40] Jorgensen L.J., Ellis G.D., Ruddell E. (2013). Fear Perceptions in Public Parks: Interactions of Environmental Concealment, the Presence of People Recreating, and Gender. Environ. Behav..

[41] Gardner P.J. (2011). Natural neighborhood networks—Important social networks in the lives of older adults aging in place. J. Aging Stud..

[42] Chaudhury H., Mahmood A., Michael Y.L., Campo M., Hay K. (2012). The influence of neighborhood residential density, physical and social environments on older adults’ physical activity: An exploratory study in two metropolitan areas. J. Aging Stud..

[43] Laatikainen T., Haybatollahi M., Kyttä M. (2018). Environmental, Individual and Personal Goal Influences on Older Adults’ Walking in the Helsinki Metropolitan Area. Int. J. Environ. Res. Public Health.

[44] Antonucci T.C. (2001). Social relations: An examination of social networks, social support, and sense of control. BT—Handbook of the Psychology of Aging.

[45] Fiori K.L., Windsor T.D., Pearson E.L., Crisp D.A. (2013). Can positive social exchanges buffer the detrimental effects of negative social exchanges? Age and gender differences. Gerontology.

[46] Hanson S., Jones A. (2015). Is there evidence that walking groups have health benefits? A systematic review and meta-analysis. Br. J. Sports Med..

[47] Hartig T., Kahn P.H. (2016). Living in cities, naturally. Science.

[48] Lin B.B., Fuller R.A., Bush R., Gaston K.J., Shanahan D.F. (2014). Opportunity or Orientation? Who Uses Urban Parks and Why. PLoS ONE.

[49] Moran M., Van Cauwenberg J., Hercky-Linnewiel R., Cerin E., Deforche B., Plaut P. (2014). Understanding the relationships between the physical environment and physical activity in older adults: A systematic review of qualitative studies. Int. J. Behav. Nutr. Phys. Act..

[50] Borst H.C., Miedema H.M.E., de Vries S.I., Graham J.M.A., van Dongen J.E.F. (2008). Relationships between street characteristics and perceived attractiveness for walking reported by elderly people. J. Environ. Psychol..

[51] Gascon M., Zijlema W., Vert C., White M.P., Nieuwenhuijsen M.J. (2017). Outdoor blue spaces, human health and well-being: A systematic review of quantitative studies. Int. J. Hyg. Environ. Health.

