# Supplementary material for: Cognitive and Sensory Dimensions of Older People’s Preferences of Outdoor Spaces for Walking: A Survey Study in Ireland
Source: Int J Environ Res Public Health. 2019 Apr 14;16(8):1340. doi: 10.3390/ijerph16081340 (PMC6518375; doi:10.3390/ijerph16081340)
Supplement: Supplementary file 1 [file ijerph-16-01340-s001.zip › CognitionWalkingAging_SupplFile1_EthicsApproval.docx]

Supplementary File 1 – Ethical Approval Letter
