# Supplementary material for: Cognitive and Sensory Dimensions of Older People’s Preferences of Outdoor Spaces for Walking: A Survey Study in Ireland
Source: Int J Environ Res Public Health. 2019 Apr 14;16(8):1340. doi: 10.3390/ijerph16081340 (PMC6518375; doi:10.3390/ijerph16081340)
Supplement: Supplementary file 1 [file ijerph-16-01340-s001.zip › CognitionWalkingAging_SupplFile2_QualitativeThemes.docx]

Supplementary File 3 – Qualitative themes and Interview Schedule

Qualitative Themes

*Theme 1: Diversity of walking purposes*

The reasons for walking outdoors in our sample can be categorised along two dimensions: a) recreational walking, which included exercising, walking a pet, and/or engaging in social interactions; b) transportation walking (e.g., shopping, banking). Both in the focus groups and interviews, the purpose of the walk appeared to influence the choice of outdoor environment: Some participants, particularly those living in urban areas, indicated that they would walk in their neighbourhood or local area for transportation, but use the car or public transport to reach other destinations where to walk for recreation (e.g., park, beach).

*“If I go for coffee or to the shops, I walk in the town but if I walk for exercise, I need to be somewhere like the beach” (WI4)*

The opposite pattern was noted for participants in less urbanised or rural areas, whereby recreational walking would occur in the area surrounding the house and transportation walking would often depend on using the car to reach places outside the neighbourhood. Overall, participants indicated that walking outdoors contributed to their health and wellbeing, but those who walked more frequently for recreation than transportation described the health benefits of walking outdoor to a greater extent.

*Theme 2: Stimulation adjustments needs*

An aspect that emerged strongly, particularly in the interviews was that participants preferred to have access to different types of outdoor environments according to their sensory and cognitive needs, which appeared to vary along a spectrum going from stimulation-seeking to stimulation-avoidance. Participants indicated a preference for walking in urban areas (e.g., city centre) because they provide variety and novelty, thus addressing the need for stimulation. Interestingly, urban walks were described as “busy” or linked to social situations such as meeting other people; however, cities were also seen as places that can cause mental overload and stress, which influenced the frequency or duration of the walk:

*"I like the busyness of the cities but I am not sure I would like to live in the centre of the city" (WI4)*

*“I’d be much more conscious of where I was and my bag but here it’s relaxed and the pollution and congestion, you know the road traffic [in the city]. Although we hear traffic here [rural area], it’s slow.” (WI3)*

On the other hand, walking in nature was deemed useful to reduce cognitive overload and relief from stress, and therefore was a way of balancing the amount of stimulation received in the city:

*“This [the beach] has to help you. When you look at this and the wind in your face, it helps your mind, you come back kind of renewed” (WI6)*

*“It’s easier to connect with nature when there are no distractions, really, yeah” (FG2)*

Participants who walked frequently in nature described their walks as enriching multisensory experiences and as a way to promote efficient cognitive processing:

*"I might be able to solve problems, my thinking process… you know, I might work through things" (WI2)*

Together with green spaces, participants also mentioned the mental benefits of walking near blue spaces, which were described as soothing and calming. Many of the participants in our sample had access to a car, thus a number of them described regular trips to the seaside to have a recreational walk.

Having access to green spaces in semi-urbanised areas was seen by many participants as an optimal solution to walking. This perspective emerged particularly among participants living and walking in towns:

*“It’s nice to get down. It’s nice to feel that there are houses around you. You know, so you’re not in an isolated area. But even, if you can see here, you still have greenery and trees, so it’s not a concrete jungle” (WI3)*

*Theme 3: Personal attitudes towards outdoor spaces*

The variety in need for stimulation adjustments described in Theme 2 appeared to depend also on individual differences in attitudes towards the outdoor environment; personal attitudes determined not only where our participants walked (e.g., nature vs. city) but also whether they walked alone or in company and the type of interaction they had with their physical surroundings. Participants who showed a high sense of biophilia (i.e., attachment to nature) or nature connectedness tended to seek natural spaces, enjoyed walking alone, and were more aware of their physical surroundings while walking:

*“To kind of get away from your own worries, you like to take things in and notice that there is a distraction by looking around" (WI1)*

Walking with others was also seen by some participants, especially those interested more in natural walks, as taking partly away from the enjoyment of the walk. One participant described taking part in walking groups as a way to visit new natural places but reported some level of annoyance at having to be with a group of people. In line with this, crowding was described by some participants as causing avoidance of certain outdoor places:

*“If the summer was very busy—we were talking earlier about beaches—we’d go over to [place name], which is a little cove between here and [place name], near [place name]. That would be a quiet beach” (WI3)*

Some participants were instead less interested and aware of their surroundings, mainly because they viewed walking as a social activity; as a consequence, they preferred being in company rather than alone:

*“I’m just one of those people that I like company. I like being with people. Perhaps I don’t do great on my own. I don’t like being on my own” (WI6)*

Personal attitudes were in some cases linked with the individual’s history and life events. Participants who had been used to walking in a certain outdoor space throughout their lives attached personal meaning to the walk and would report positive affective experiences when walking. For instance, one participant mentioned that meeting a certain flower along her walk in the woods would remind her of her father, who used to take her there as a child. Changes in life were also described as contributing to how people viewed walking outdoors:

*“The thing is now that I’m widowed and I’m on my own and it took me quite a while to learn to do things on my own. Like walking can be very boring on your own, you know. But now I find I don’t mind it at all, you know. And I look at things and I meet people and people know me.” (WI4)*

Furthermore, attitudes towards sensation-seeking/avoidance influenced the participants’ willingness to undertake new and diverse walks. Sensation-seekers reported higher frequency of visiting new places. On the other hand, some participants described a need for familiarity with the places where they walked:

*“If I was on my own no. I’d get lost in a saucer. Not familiar places but in a strange place. I wouldn’t be very confident of finding my way back…taking account of the landmarks going in” (WI5)*

*“You have an idea of the geography of a place and know how to get about” (WI7)*

Therefore, familiarity with a place appeared to facilitate spatial orientation and promote comfort during walking, as well as mitigating concerns about safety.

*Theme 4: Social dimensions of walking outdoors*

Perceived safety was an important contributor to how comfortable participants felt when outdoors and where they would choose to walk. Concerns about social safety (e.g., being attacked or robbed) were expressed both for urban and natural spaces:

*"Being on my own walking in a city, my antennae would be up. You’d be more mindful. " (WI3)*

*“Sometimes if I’m deep down in the woods on my own, I might feel anxious” (WI2)*

Therefore, nature was associated with feelings of being isolated and potentially vulnerable to unpleasant circumstances; at the same time, being in a busy place such as the city meant being exposed to potentially unsafe social circumstances. Participants showed diversity of views on the presence of people when walking: Walking in places used by other people appeared to promote perceived safety for some participants, who saw being around others as a way to feel less isolated and also engaging in social interactions; thus, the social dimension of being outdoors was important for some participants, particularly those who took part in active retirement groups preferred to walk in company rather than alone:

*“I’d go with somebody likeminded that is in the group. They like walking, you like walking along with them” (FG1)*

However, other participants described having people around while walking as a potential source of stress, particularly in crowded situations: These negative feelings were associated with concerns about safety (i.e., meeting someone undesirable), as noted above, but also with fears of experiencing hazardous situations such as bumping into other people or being hit by a cyclist.

*Theme 5: Environment physical attributes*

This theme was related to factors of walkability (accessibility and design), perceived stressors and structural hazards.

Accessibility of places suitable for walking was seen as crucial by most participants, particularly in the focus groups. Accessing a variety of outdoor spaces was facilitated by the use of a car or public transport, which most of our participants used. Participants living in less urbanised areas felt that their dependence on the car could affect their ability to access favourite places when unable to drive:

*“When you think about it, I mean, if you are living in an urban area and you don’t have transport, how do you get out to the country to a place like this? So that would be a huge deterrent, wouldn’t it, if you didn’t have transport?” (WI2)*

On the other hand, participants who lived in suburban areas of the city discussed the increasing availability of services and amenities in their local community over the years as a positive influence on walking, because it afforded less dependence on the car:

*“You need never be going to the city…It’s the convenience of everything, the school, churches, shops” (FG2)*

Together with being accessible, outdoor spaces were perceived as facilitating walking if providing adequate infrastructure and being aesthetically appealing:

*“Well, specifically for walking, such as the [place name], there is a pedestrian pathway. There is an attractive environment, yes.” (WI7)*

Related to walkability, the quality of road pavements was felt as a barrier to walking, especially by participants who were older and/or using walking aids, who felt that poor pavements contributed to their fear of falling and thus led to avoiding certain places for walking. Traffic was described both as a hazard and as a source of stress. Participants felt that walking in spaces with high volumes of traffic required more mental effort in order to stay safe:

*“You need all your focus because here now like they’re coming in four directions” (WI5)*

Some participants identified traffic as a negative influence on the ability to use and enjoy local outdoor spaces:

*“And [neighbourhood name] was a little village and the village is gone really. We used to have a community thing in [neighbourhood name]. But the traffic is horrendous.” (FG1)*

Notably, participants using urban outdoor spaces were less bothered by traffic, feeling that it was part of normal urban life and that it was a temporary part of the walk. However, noise was felt as a major perceptual stressor which participants associated with traffic; along this, quietness was seen as something that participants sought in their walks, particularly those who tended to use more natural outdoor spaces. Interestingly, some participants avoided using certain outdoor spaces at times of the day when there was more traffic (e.g., when children got out of school), showing adaptation to the surrounding environments in order to maintain walking habits.

Interview schedule

The schedule was used as a general guide and was adapted for the focus groups and the walk along interviews

Focus group

1. Could you tell me about the types of outdoor places you like to use for walking?
2. Do you ever try different routes? Why/why not?
3. How do you feel when using an outdoor space that you like?
4. How do you feel about (*any of*) traffic/noise/having other people around/green spaces/variety in the places where you walk?
5. How do you think that going to that outdoor space affects your wellbeing and health?

Interviews

1. Could you tell me about this walk that we are doing together?
2. What do you like about this walk?
3. Do you ever try a different route? Why/why not?
4. How do you feel when walking along this route?
5. How do you feel about (*any of*) traffic/noise/having other people around/green spaces/variety in the places where you walk?
6. How do you think that walking along this route affects your wellbeing and health?
