# Supplementary material for: Cognitive and Sensory Dimensions of Older People’s Preferences of Outdoor Spaces for Walking: A Survey Study in Ireland
Source: Int J Environ Res Public Health. 2019 Apr 14;16(8):1340. doi: 10.3390/ijerph16081340 (PMC6518375; doi:10.3390/ijerph16081340)
Supplement: Supplementary file 1 [file ijerph-16-01340-s001.zip › CognitionWalkingAging_SupplFile3_SampleCharacteristics.docx]

Supplementary File 3 – Survey Sample Characteristics

| *Sample characteristics* | |  |  |  |  |
| --- | --- | --- | --- | --- | --- |
| Measure | Mean | SD | Median | IQR | Range |
| Sensory sensitivity | 30.57 | 8.28 | 31.00 | 11.00 | 11-55 |
| Cognitive failures | 31.66 | 13.35 | 31.00 | 16.50 | 0-68 |
| Neighbourhood appeal | 13.19 | 2.14 | 14.00 | 3.00 | 6-15 |
| Structural quality | 10.22 | 3.75 | 11.00 | 6.25 | 3-15 |
| Perceptual stressors (reversed) | 10.11 | 3.68 | 11.00 | 5.50 | 3-15 |
| Accessibility | 6.75 | 3.29 | 8.00 | 7.00 | 2-10 |
| Sense of community | 4.15 | 1.02 | 4.00 | 1.00 | 1-5 |
| Variety | 3.68 | 1.14 | 4.00 | 2.00 | 1-5 |
| Quietness | 3.66 | 1.13 | 4.00 | 2.00 | 1-5 |
| Green spaces | 4.20 | 0.98 | 4.00 | 1.00 | 1-5 |
| People | 3.30 | 1.27 | 3.00 | 2.00 | 1-5 |
| *Notes*. IQR = Interquartile range; SD = Standard Deviation | | | | | |
