# Supplementary material for: Cognitive and Sensory Dimensions of Older People’s Preferences of Outdoor Spaces for Walking: A Survey Study in Ireland
Source: Int J Environ Res Public Health. 2019 Apr 14;16(8):1340. doi: 10.3390/ijerph16081340 (PMC6518375; doi:10.3390/ijerph16081340)
Supplement: Supplementary file 1 [file ijerph-16-01340-s001.zip › CognitionWalkingAging_SupplFile4_RegressionAnalyses.docx]

**Supplementary File 4**

Regression analyses – Walking preferences by cognitive/sensory measures and urbanity level

**Variety**

|  | **Cognitive Failures** | | | **Sensory Sensitivity** | | |
| --- | --- | --- | --- | --- | --- | --- |
| **Measure** | **Prop OR** | **P-value** | **Wald chi2** | **Prop OR** | **P-value** | **Wald chi2** |
| Cognitive failures by urbanity level |  |  | 1.32 |  |  | 3.98 |
| Inner city | 0.99 | .60 |  | 1.05 | .11 |  |
| City suburbs | 1.00 | .94 |  | 1.04 | .08 |  |
| Town | 1.01 | .74 |  | 1.06 | .05 |  |
| Village | 1.01 | .79 |  | 1.05 | .17 |  |
| Countryside | 0.99 | .66 |  | 1.05 | .13 |  |
| Female | 0.91 | .82 | 0.05 | 1.13 | .76 | 0.09 |
| Age | 1.01 | .99 | .74 | 0.98 | .52 | 0.42 |
|  |  |  |  |  |  |  |
| Goodness-of-fit Pearson’s chi2 | 328.29 |  |  | 395.27 |  |  |
| Omnibus test likelihood ration chi2 | 1.64 | .97 |  | 4.16 | .76 |  |
| Pseudo *R2* | 0.01 |  |  | 0.02 |  |  |
| *Notes*. Chi2 = Chi-squared; Female refers to the Gender variable, with female participants compared to male participants; Prop OR = Proportional Odds Ratio. Model 2 controls for gender and age.  Wald chi2 refers to the test of model effects. Statistical significance for this test is shown as * p < .05, ** p < .01, *** p < .001. | | | | | | |

**Quietness**

|  | **Cognitive Failures** | | | **Sensory Sensitivity** | | |
| --- | --- | --- | --- | --- | --- | --- |
| **Measure** | **Prop OR** | **P-value** | **Wald chi2** | **Prop OR** | **P-value** | **Wald chi2** |
| Cognitive failures by urbanity level |  |  | 5.38 |  |  | 5.89 |
| Inner city | 0.99 | .65 |  | 1.05 | .05 |  |
| City suburbs | 0.97 | .11 |  | 1.02 | .35 |  |
| Town | 0.98 | .29 |  | 1.04 | .16 |  |
| Village | 1.01 | .54 |  | 1.05 | .13 |  |
| Countryside | 0.98 | .42 |  | 1.04 | .18 |  |
| Female | 0.51 | .12 | 2.38 | 0.73 | .43 | 0.61 |
| Age | 1.02 | .36 | 0.84 | 1.01 | .55 | 0.35 |
|  |  |  |  |  |  |  |
| Goodness-of-fit Pearson’s chi2 | 337.38 |  |  | 354.75 |  |  |
| Omnibus test likelihood ration chi2 | 6.91 | .44 |  | 6.72 | .46 |  |
| Pseudo *R2* | 0.03 |  |  | 0.03 |  |  |
| *Notes*. Chi2 = Chi-squared; Female refers to the Gender variable, with female participants compared to male participants; Prop OR = Proportional Odds Ratio. Model 2 controls for gender and age.  Wald chi2 refers to the test of model effects. Statistical significance for this test is shown as * p < .05, ** p < .01, *** p < .001. | | | | | | |

**Green spaces**

|  | **Cognitive Failures** | | | **Sensory Sensitivity** | | |
| --- | --- | --- | --- | --- | --- | --- |
| **Measure** | **Prop OR** | **P-value** | **Wald chi2** | **Prop OR** | **P-value** | **Wald chi2** |
| Cognitive failures by urbanity level |  |  | 6.78 |  |  | 4.36 |
| Inner city | 0.97 | .08 |  | 1.01 | .83 |  |
| City suburbs | 0.99 | .68 |  | 1.02 | .55 |  |
| Town | 0.98 | .57 |  | 1.02 | .47 |  |
| Village | 0.98 | .53 |  | 1.01 | .74 |  |
| Countryside | 1.02 | .38 |  | 1.06 | .09 |  |
| Female | 1.01 | .97 | 0.01 | 1.18 | .69 | 0.15 |
| Age | 0.96 | .14 | 2.18 | 0.96 | .11 | 2.58 |
|  |  |  |  |  |  |  |
| Goodness-of-fit Pearson’s chi2 | 447.13 |  |  | 425.52 |  |  |
| Omnibus test likelihood ration chi2 | 9.16 | .24 |  | 7.18 | .41 |  |
| Pseudo *R2* | 0.04 |  |  | 0.07 |  |  |
| *Notes*. Chi2 = Chi-squared; Female refers to the Gender variable, with female participants compared to male participants; Prop OR = Proportional Odds Ratio. Model 2 controls for gender and age.  Wald chi2 refers to the test of model effects. Statistical significance for this test is shown as * p < .05, ** p < .01, *** p < .001. | | | | | | |
